# The effects of a psychological intervention directed at optimizing immune function: study protocol for a randomized controlled trial

**DOI:** 10.1186/s13063-017-1983-7

**Published:** 2017-05-26

**Authors:** Lemmy Schakel, Dieuwke S. Veldhuijzen, Henriët van Middendorp, Corine Prins, Simone A. Joosten, Tom H. M. Ottenhoff, Leo G. Visser, Andrea W. M. Evers

**Affiliations:** 1 Faculty of Social and Behavioural Sciences, Institute of Psychology, Health, Medical and Neuropsychology Unit, Leiden University, PO Box 9555, 2300 RB Leiden, The Netherlands; 2 Leiden Institute for Brain and Cognition, Leiden University, Leiden, The Netherlands; 3 Department of Infectious Diseases, Leiden University Medical Centre, Leiden, The Netherlands; 4 Department of Psychiatry, Leiden University Medical Centre, Leiden, The Netherlands

**Keywords:** BCG vaccination, Immune system, e-Health, Cognitive behavioral therapy, Serious gaming, Psychophysiology, Stress, Cytokines

## Abstract

**Background:**

Previous research has provided evidence for the link between psychological processes and psychophysiological health outcomes. Psychological interventions, such as face-to-face or online cognitive behavioral therapy (CBT) and serious games aimed at improving health, have shown promising results in promoting health outcomes. Few studies so far, however, have examined whether Internet-based CBT combined with serious gaming elements is effective in modulating health outcomes. Moreover, studies often did not incorporate psychophysiological or immunological challenges in order to gain insight into physiological responses to real-life challenges after psychological interventions. The overall aim of this study is to investigate the effects of a psychological intervention on self-reported and physiological health outcomes in response to immune and psychophysiological challenges.

**Methods/design:**

In a randomized controlled trial, 60 healthy men are randomly assigned to either an experimental condition, receiving guided Internet-based (e-health) CBT combined with health-related serious gaming elements for 6 weeks, or a control condition receiving no intervention. After the psychological intervention, self-reported vitality is measured, and participants are given an immunological challenge in the form of a *Mycobacterium bovis* Bacillus Calmette-Guérin (BCG) vaccination. One day after the vaccination, participants are asked to perform several psychophysiological tasks in order to explore the effects of the psychological intervention on participants’ stress response following the immune challenge. To assess the delayed effects of vaccination on self-reported and physiological health outcomes, a follow-up visit is planned 4 weeks later. Total study duration is approximately 14 weeks. The primary outcome measure is self-reported vitality measured directly after the intervention. Secondary outcome measures include inflammatory and endocrine markers, as well as psychophysiological measures of heart rate and skin conductance in response to the psychophysiological tasks after the BCG vaccination.

**Discussion:**

The innovative design features of this study – e.g., combining guided e-health CBT with health-related serious gaming elements and incorporating immunological and psychophysiological challenges – will provide valuable information on the effects of a psychological intervention on both self-reported and physiological health outcomes. This study will offer further insights into the mechanisms underlying the link between psychological factors and health outcomes and is anticipated to contribute to the optimization of health care strategies.

**Trial registration:**

Nederlands Trial Register, NTR5610. Registered on 4 January 2016.

**Electronic supplementary material:**

The online version of this article (doi:10.1186/s13063-017-1983-7) contains supplementary material, which is available to authorized users.

## Background

The conventional way to reduce inflammation is to administer anti-inflammatory pharmaceutical agents such as corticosteroids or nonsteroidal anti-inflammatory drugs. These drug treatments, however, often have (severe) side effects or are nonspecific [1–4]. Previous research has provided evidence for a link between psychological processes and inflammatory responses [5, 6]. Psychological interventions aimed at reducing inflammatory processes without the use of pharmaceutical agents could, therefore, be useful to supplement, or even (partially) replace current drug treatments. For this reason, it is important to increase our understanding of the effectiveness of psychological interventions on health outcomes. However, research on this topic is still in its infancy.

A meta-analysis focusing on the effects of several psychological interventions (e.g., relaxation, conditioning, stress management, hypnosis, and disclosure interventions) on immune-related health outcomes has demonstrated that psychological interventions can modulate certain features of the immune response; these modulations are reflected in lower proinflammatory and/or higher anti-inflammatory responses [7]. Overall, the meta-analysis provides modest evidence that psychological interventions affect immune function. In light of these findings, it is important to acquire a better understanding of the effects of psychological interventions on self-reported and physiological health outcomes, and of the mechanisms involved.

Internet-based cognitive behavioral therapy (e-health CBT) is an upcoming and innovative tool that may improve the effectiveness of psychological interventions. E-health CBT has been shown to be effective in decreasing psychological stress in various clinical populations, including patients with cancer, chronic pain, and irritable bowel syndrome [8–10]. In the area of somatic conditions, a meta-analysis by van Beugen and colleagues (2014) showed that e-health CBT was effective: the effects were comparable with face-to-face CBT [11]. Furthermore, some studies have provided evidence that using e-health CBT results in cost savings compared with face-to-face CBT [12–14]. Finally, e-health CBT can be more convenient and flexible than face-to-face therapy, and reduces travelling time [15].

An important part of a CBT intervention is to strengthen the effects of explicit behavior-change techniques. To increase the effects, the underlying cognitive processes can be further trained by means of specific cognitive behavioral strategies. Such strategies include principles of reward and evaluative conditioning [16], as can be applied in serious gaming. Recent studies show that the addition of serious gaming elements to e-health interventions can improve knowledge transfer. Moreover, due to the entertainment aspect, adding serious gaming elements can help to overcome motivational barriers [17]. Serious games have proved effective in improving knowledge and self-management skills in young people with chronic conditions [18], for example, as well as in increasing knowledge about drug and alcohol use in adolescents [19]. Serious games can also have beneficial effects on factors that are considered important for a healthy lifestyle in general such as healthy food choices and physical activity [17]. So far, serious games have not often been investigated in combination with CBT. A study in bulimia patients and a study in patients with a severe gambling disorder have shown better therapeutic outcomes when CBT interventions are complemented with serious gaming than in the case of a standalone CBT intervention [20, 21]. Thus, serious games may be a promising add-on to CBT, as an innovative tool to motivate users to increase their knowledge and skills regarding health-related behavior.

Most studies on the effects of psychological interventions have focused on basal health outcomes (e.g., general levels of psychological and immune functioning). The aim of psychological interventions is to enhance an individual’s ability to cope with physical and psychological stressors and daily life hassles, which can presumably best be studied by evoking real-life challenges such as immune challenges (i.e., inflammatory reactions) and psychophysiological challenges (i.e., stress responses). Inflammatory reactions can be elicited experimentally by, for example, in vivo or in vitro stimulation of the immune system; stress responses can be elicited by having participants perform specific psychophysiological tasks. In a previous study, a relatively short-term inflammatory response was induced by using an in vivo stimulation with lipopolysaccharide (LPS). The researchers demonstrated that a physical exercise and breathing intervention directed at optimizing immune functioning showed promising effects when compared to no intervention [22]. After the LPS stimulation, the participants who had received the intervention exhibited significantly lower proinflammatory cytokine levels and fewer flu-like symptoms than the control group. Another study evaluated the effects of a stress management training on immune outcomes in patients with rheumatoid arthritis. In response to a potent psychosocial stressor, the group of participants who had received stress management training showed altered cortisol and interleukin (IL)-8 levels compared to the group who had not received training [23, 24].

A relatively safe method to stimulate the immune system in vivo is to use a vaccine as an immunological challenge. One such live vaccine with a good safety record in children and adults, which is routinely administered to infants in many countries all over the world, is the *Mycobacterium bovis* Bacillus Calmette-Guérin (BCG) vaccine, the common vaccine against tuberculosis (TB) [25]. Furthermore, BCG vaccination induces a proinflammatory cytokine response [26]. Therefore, BCG vaccination seems an appropriate immunological challenge in order to investigate immune reactivity after a psychological intervention. To obtain more insight into the effects of a psychological intervention on the responsiveness to stress, participants were additionally exposed to relevant psychophysiological tasks after receiving a BCG vaccination [27–29].

Based on the theoretical background and empirical findings, a two-armed randomized controlled trial (RCT) has been designed to investigate the effects of a psychological intervention directed at optimizing immune function. We aim to assess the effects of the psychological intervention on self-reported and physiological health outcomes in response to immune and psychophysiological challenges. In this RCT, participants are randomly allocated to an experimental or a control condition. The participants in the experimental condition receive guided e-health CBT in combination with health-related serious gaming elements, whereas the controls receive no interventions. We expect, first, that after the intervention and vaccination participants in the experimental condition will show higher self-reported vitality (as measured by a composite score of vitality and fatigue) compared to the control condition. Second, we expect that participants in the experimental condition will show optimized health outcomes on the psychological and physiological variables assessed after BCG vaccination and the psychophysiological tasks. Below, we describe the study protocol.

## Methods/design

### Study design

**Fig. 1:**
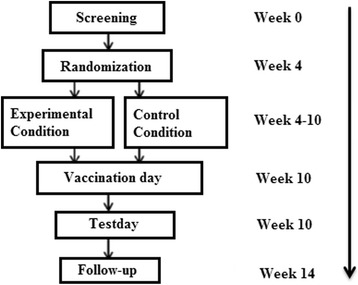
Flowchart of the study design

In order to investigate whether a psychological intervention, consisting of a guided e-health CBT in combination with health-related serious gaming elements, can modulate self-reported and physiological health outcomes in healthy participants, an RCT will be conducted. The study has been approved by the Medical Ethical Committee of the Leiden University Medical Center (registration number P15.099/NL52434.058.15). The study will be conducted in accordance with the Declaration of Helsinki and the International Conference on Harmonization (ICH) Guidelines on Good Clinical Practice (GCP). Figure 1 shows the flowchart of the study design and the Standard Protocol Items: Recommendations for Interventional Trials 2013 (SPIRIT) Checklist is presented as Additional file 1.

### Procedures

Participants are recruited from the Leiden University student population via local digital or printed advertisements. Testing takes place at the Leiden University Medical Center in The Netherlands. After providing informed consent to the test leader, participants are first screened for potential physical and psychiatric conditions that might interfere with their safety or with the study protocol. Participants who meet the inclusion criteria (see below for details) are randomized to the experimental or the control condition. Participants in the experimental condition receive a psychological intervention for 6 weeks; participants in the control condition do not receive any intervention during this period. Subsequently, all participants are given a BCG vaccination to challenge the immune system in vivo. One day after vaccination, participants take part in a test day, on which they perform psychophysiological tasks. Four weeks after the test day, finally, a follow-up session is planned in order to evaluate the effects of the psychological intervention on health outcomes in the longer term. The schedule of activities in the study is presented in Table 1.

**Table 1:** Schedule of study activities

		Study period				
Visit	Screening	Allocation	Intervention period	Vaccination day	Test day	Follow-up
Time point	Week 0	Week 4	Weeks 4–10	Week 10	Week 10	Week 14
Enrolment:						
Eligibility screen	X					
Informed consent	X					
Allocation		X				
Interventions:						
Intervention condition			X			
Control condition			X			
Assessments:						
Serum blood sample	X			X	X	X
Heparin blood sample	X			X	X	
Medical screening questionnaire	X					
MINI Psychiatric Interview	X					
IGRA test (interferon-gamma release assay)	X					
HIV test	X					
Vaccination				X		
Psychophysiological stress tasks					X	
Saliva samples	X			X	X	X
Heart rate, heart rate variability and skin conductance levels	X				X	X
Questionnaires	X			X	X	X

### Study population, randomization, and blinding

Eligibility is assessed by junior researchers in collaboration with a clinical psychologist and a study nurse in collaboration with a physician; the specific inclusion and exclusion criteria are described below. We aim to include 60 healthy participants between 18 and 35 years of age. Since the menstrual cycle is known to have an effect on immune function [30], only men are included in this study. Further inclusion criteria are having a good understanding of written and spoken Dutch, and being naive for TB. Pre-existing immunity against *Mycobacterium tuberculosis* is actively screened by performing the Quantiferon TB-Gold™ test on all participants at the screening visit; only participants who test negative are included in the study. Moreover, an HIV test is performed for all participants, as infection with HIV may be a contraindication for vaccination with live vaccines such as BCG. Participants are furthermore excluded from the study if they: (1) have a history of inflammatory or cardiovascular diseases, (2) are allergic to any of the vaccine components, (3) have a history of exposure to open TB, (latent) TB disease, or treatment for TB, (4) have undergone a BCG vaccination at any time prior to entering the trial, (5) have received another live vaccination 4 weeks or less prior to the BCG vaccination, (6) have been treated with immune-modulating drugs 3 months or less prior to enrollment, (7) have (of have had) a disease affecting the lymphoid organs, (8) are known to have congenital or acquired immune deficiencies, (9) have psychiatric (*Diagnostic and Statistical Manual of Mental Disorders, version 5*; DSM-5) or somatic conditions that interfere with their safety and/or the study protocol, (10) are professional sports players or perform extreme exercise, (11) have a history of excessive drinking or drug use, (12) are actively participating in other clinical trials, or (13) do not give consent for their general practitioner to be informed of their BCG vaccination.

In order to balance the allocation equally during each season, since season is associated with the prevalence rate of influenza [31], a block randomization is performed, with block sizes of 4. Block randomization is generated by the first author using an online random number generator (http://www.random.org). In order to avoid possible expectancy effects, the test leader on the test day is blinded to the group allocation of the participants. Participants are aware of allocation since they have to be informed that they have been randomized to a condition that includes a psychological intervention or a condition that includes no intervention.

Anonymized participant identification codes are used to link data to participants. Study personnel and the person who conducts the data monitoring are the only people who have access to the personalized data forms.

### Psychological intervention

Participants allocated to the experimental condition receive guided e-health CBT. Immune function is known to be influenced by behavioral and lifestyle factors such as healthy food and exercise, relaxation and sleep, and cognitions and worldview [24, 32–36]. Therefore, these factors are taken into account in this intervention. All modules are based on evidence-based interventions in this area [37, 38]. A therapist, referred to as an e-Coach, guides participants through the online environment by giving homework assignments, following the progress of participants, and sending motivational feedback messages. The intervention consists of six modules and starts with a face-to-face intake interview between the e-Coach and the participant. This interview serves as an introduction to the online intervention and to set short-term and long-term goals, based on the various topics of the individual modules of the intervention (module 1). Modules 2 to 5 focus on lifestyle: healthy food and exercise, relaxation, sleep, and cognitions and worldviews. Each module contains approximately ten online assignments such as relaxation exercises. At the start of each module, participants make a plan for the week in which they describe how they aim to reach their goals. Participants fill out a daily diary to keep track of their progress towards their goals and the activities that they have undertaken to reach them, and to reflect on wellbeing and sleep quality. At the end of each module, participants receive a summary of what they have learned during the module and are required to reflect on the extent to which they have reached their goals. The intervention ends with a module that focuses on setting long-term goals and preventing relapse.

In addition to the online intervention, participants in the experimental condition play a serious game called ViaNova© to optimize immune function; the game has been developed in a collaboration between Leiden and Delft Universities. Before starting the game, participants design an avatar that serves as a representation of their ideal self. Furthermore, the game includes a coach (mirroring the e-Coach of the online intervention) who navigates the avatar through the game. The serious game contains several mini-games based on four themes matching the modules of the online intervention, including lifestyle factors involving healthy food and exercise, relaxation, sleep, and cognitions and worldview; there are four different rooms in the game, with each room reflecting one of these themes. The games are all health-related, based on cognitive behavioral strategies including principles of reward and evaluative conditioning. An example of a game is the approach-avoidance task [39], in which participants have to pull healthy items towards them and push unhealthy items away by clicking on the corresponding arrows on the keyboard. Participants are instructed to play various games 5 days a week, throughout the 6 weeks of the intervention.

### Vaccination day

After the 6-week period, all participants complete questionnaires about the primary and secondary outcomes. Furthermore, a blood sample and a saliva sample are taken, and participants are vaccinated with the live-attenuated BCG by a trained research nurse, through intradermal injection in the upper arm. To monitor for possible side effects, participants fill in a diary for 4 weeks after the vaccination.

### Assessments on the test day

One day after the vaccination, participants complete three different psychophysiological tasks: a modified version of the PASAT [29], the CPT [28], and the TSST [27].

#### Modified Paced Auditory Serial Addition Task (PASAT)

The PASAT was originally developed as a measure for information processing speed [40]. In this task, participants are presented with a series of single-digit numbers which are delivered through an audio player. The participants’ task is to add each number to the number presented previously [40]. The task consists of two parts, separated by a 1-min break, in which four consecutive 2-min series of digits are presented at different intervals at an increasing pace. To induce psychological stress during this task, we present participants with a modified version, based on a version used in previous studies [29, 41]. In this task, participants are exposed to an aversive noise if they give an incorrect response. Furthermore, they are instructed to watch their own face on a computer screen during the task and are informed that these recordings will be analyzed by a body language expert. Previous research has shown that this version of the PASAT can modulate cardiovascular responses, in that stress-induced hemoconcentration appeared [29]. Furthermore, in a previous study investigating the effects of vaccine-induced inflammation on mental stress levels, cardiovascular responses to this version of the PASAT were attenuated by vaccination [42].

#### Cold Pressor Test (CPT)

In order to induce nonharmful and quickly reversible physical stress, participants are exposed to a CPT [28]. Participants are instructed to place their dominant hand in a tank of cold water, at a temperature of about 2 °C (±0.1 °C), until immersion becomes unbearable. The maximum immersion time is 4 min, but the participants are not aware of this time limit. The pain threshold (first moment of pain sensation) and maximum immersion time are recorded.

#### Trier Social Stress Test (TSST)

Participants are exposed to the TSST [27, 43], a standardized laboratory stress task, consisting of a mock job interview and mental arithmetic task in front of a two-member jury. First, participants are given 5 min to prepare a presentation about their ‘dream’ job position. Subsequently, participants present in front of a two-member jury while being recorded by a video-camera and voice recorder. During the presentation, the jury members take notes and ask some questions without providing feedback to the participants. After 5 min, participants are instructed to count backwards in steps of 17 from 1965 to 0. When participants make a mistake or do not answer fast enough, they are told to start at 1965 again. The total duration of the TSST is approximately 15 min [27]. This task has been found to be sensitive to inducing inflammatory responses [44], as well as neuroendocrine and autonomic nervous system responses [27].

### Follow-up

Four weeks after the vaccination and test day, a follow-up session is planned in order to evaluate the effects of the psychological intervention on health outcomes in the longer term. During the follow-up session, participants fill in questionnaires. Furthermore, a blood sample and a saliva sample are taken, and heart rate and skin conductance are measured at rest.

### Self-report outcome measures

Participants fill out several questionnaires at baseline, on the day of vaccination, on the test day, and at the follow-up.

Vitality is measured by a composite of the Subjective Vitality Scale – State version (SVS) [45], and the (reverse-scored) Checklist Individual Strength (CIS-20) [46]. The SVS has been validated in a student population as a vitality measure with a good internal consistency and reliability [45, 47]. The CIS-20 was developed to measure fatigue severity [48] and has a good internal consistency and reliability [46]. A composite score of the SVS and CIS-20 is used as primary outcome measure.

Several other questionnaires are administered exploratively to assess their possible moderating role in the effects of a psychological intervention on self-reported and physiological health outcomes.

### Physiological outcome measures

#### Cardiovascular measures

Heart rate and skin conductance are measured with a BIOPAC MP150 system® at baseline, on the test day, and on the follow-up day.

#### Inflammatory measures

Serum blood samples are taken to measure inflammatory markers, such as cytokine levels (e.g., IL-6, Il-8), at baseline, on the day of vaccination, at the start and end of the test day, and at follow-up. A heparin blood sample is taken at baseline, on the day of vaccination, and at the start of the test day. This sample is used to stimulate blood cells with LPS in vitro in a 37 °C incubator for 6 h; the stimulated and control plasma samples are then centrifuged and stored at −80 °C. All inflammatory parameters are measured in batches, including complete follow-up samples of individual participants.

#### Endocrine measures

Saliva is taken to assess endocrine responses (e.g., cortisol, alpha-amylase) at the same time points as the serum blood samples and additionally after each stress task on the test day, 1 day after vaccination.

### Statistical analyses

#### Primary outcome

Effects of the psychological intervention on vitality are assessed in an analysis of variance with inclusion of covariates (ANCOVA) when appropriate. Vitality after the psychological intervention is used as dependent variable, and condition (experimental or control condition) is used as a between-subjects factor. Baseline measurement of vitality is used as a covariate.

#### Secondary outcomes

Inflammatory responses measured in blood at the screening, after the intervention (on the vaccination day), on the test day 1 day after vaccination, and at the follow-up session are assessed in multilevel models. The inflammatory responses are used as dependent variables, with group allocation, baseline measurements, and time serving as independent variables. Analyses for LPS-stimulated blood (measured at the screening, on the vaccination day, and the start of the test day) are conducted in a similar way.

Endocrine responses measured in saliva at screening, after the intervention (on the vaccination day), after each stress task 1 day after vaccination, and during follow-up are evaluated as dependent variables in a multilevel model, with group allocation, baseline measurement of the dependent variable, and time as independent variables. Analyses for heart rate and skin conductance are conducted in a similar way.

Demographic variables and self-reported measures are explored as possible predictors of the primary outcomes.

#### Sample size calculation

The final power calculation was based on a study examining the effects of a stress management intervention on the level of psychological distress, using a comparable design [24]. Power analysis of this study indicated that 30 participants per condition would be sufficient to detect an adjusted effect size of *f* = 0.45 on psychological distress, with a power of 0.80, and an alpha level of 0.05. For the SVS, one representative study has found an effect size of *f* = 0.44 in change in vitality at post intervention in an uncontrolled study on a walking intervention including a motivational intervention [49]; an effect size of *f* = 0.40 was found in another representative study for the difference in change in vitality between an endurance intervention group and a control group at post intervention [50]. Also, with regard to the CIS-20, similar effect sizes have been reported for cognitive behavioral interventions in, among other populations, patients with rheumatoid arthritis [51]. Therefore, a total sample size of 60 participants was deemed sufficient to identify detectable and clinically relevant differences in the outcome parameters of the current study.

## Discussion

The present study evaluates whether self-reported and physiological health outcomes can be modulated by a psychological intervention directed at optimizing immune function. The intervention consists of guided e-health CBT in combination with health-related serious gaming elements and will be tested in healthy men. The study will contribute to the findings on the effects of psychological interventions on psychophysiological stress reactivity after an immunological challenge and provide further evidence on the link between psychological and immunological mechanisms [7].

A unique feature of this study is that we use a psychological intervention based on multiple strategies (e-health CBT and serious gaming) directed at optimizing immune function. The study is among the first to use a combination of guided e-health CBT and health-related serious gaming elements. Serious gaming can be a promising add-on to e-health CBT since this highly innovative tool can strengthen skills, attitudes, and knowledge about health in an entertaining manner. The results of the present study will, therefore, provide more insight into these psychological interventions directed at optimizing immune function and their potential effectiveness on health outcomes, both self-reported and physiological. Furthermore, the advantages of e-health CBT are that both participants and therapists can use and log on to the intervention at the time and place they prefer. Therefore, the intervention developed may be easier to use, more time efficient, and consequently less expensive than traditional face-to-face therapy. Complementing guided e-health CBT with health-related serious gaming elements seems to be a promising approach to optimize health outcomes, since multiple cognitive behavioral strategies are involved. If the combined intervention of guided e-health CBT and health-related serious gaming elements turns out to be effective in modulating self-reported and physiological health outcomes, the individual components of this psychological intervention can be investigated in further studies in order to gain more insight into the effectiveness of the different components.

In addition to an innovative psychological intervention, the study design incorporates validated immunological challenges in the form of in vivo and in vitro stimulation of immune responses. A previous study incorporated a hepatitis B vaccination to investigate the effects of an emotional disclosure intervention on immune reactivity [52]. The researchers found that participants who received the emotional disclosure intervention showed higher levels of antibodies in response to the hepatitis B vaccination than the group who did not receive any intervention. Live vaccines, such as the BCG vaccine, come closer than other vaccines to eliciting the same immune response as is observed after natural infection. Consequently, by including BCG vaccination as an immunological challenge, this study will provide more insight into the real-life effects of a psychological intervention on immune function and subsequently on the development of protection against infectious diseases. Besides BCG vaccination, LPS stimulation in vitro at baseline, before vaccination, and after vaccination provides more insight into the in vitro immune reactivity that occurs in response to a psychological intervention.

The study also incorporates psychophysiological tasks. This allows us to obtain more insight into the psychological intervention’s effects on stress reactivity. Previous studies have shown that various stressors can lead to different psychophysiological stress responses. More heightened endocrine responses and anticipatory stress appraisals were found after exposure to the TSST than after exposure to the CPT [53, 54], for example. By combining multiple psychophysiological and physiological stressors, more detailed information can be gathered about the stress response following an immune challenge. In addition, this study is one of few studies to evaluate the effects of a psychological intervention on health outcomes at follow-up [23, 24]: the study design incorporates a test session 4 weeks after the intervention. By implementing in vivo and in vitro immunological challenges and psychophysiological challenges in combination with a follow-up measurement, this study has the potential to advance scientific knowledge into the mechanisms underlying the relation between psychological and immunological factors.

In conclusion, the present study design is expected to provide valuable information about the role of psychological mechanisms in optimizing health outcomes in healthy men. It will help to unravel the underlying mechanisms of psychophysiological stress reactivity to immunological challenges and may ultimately contribute to the development of new health care strategies. If it turns out that this psychological intervention can modulate various health outcomes, it can be implemented in health care and may partially or fully replace medication use. This may mean that fewer people suffer from the side effects of medication use and may also lead to reductions in health care costs.

## Trial status

This trial is currently in the participant recruitment phase.

## Additional file

Additional file 1: SPIRIT_Checklist. (DOC 254 kb)

## Abbreviations

ANCOVA: Analyses of covariance
BCG: Bacillus Calmette-Guérin
CBT: Cognitive behavioral therapy
CIS-20: Checklist Individual Strength
CPT: Cold Pressor Test
DSM-5: Diagnostic and Statistical Manual of Mental Disorders, version 5
GCP: Good Clinical Practice
ICH: International Conference on Harmonization
LPS: Lipopolysaccharide
NTR: Nederlands Trial Register
PASAT: Paced Auditory Serial Addition Task
RCT: Randomized controlled trial
SPIRIT: Standard Protocol Items: Recommendations for Interventional Trials
SVS: Subjective Vitality Scale – State version
TB: Tuberculosis
TSST: Trier Social Stress Test
